# Supplementary figures and images for: Evidence of allocentric spatial learning in male rats with large lesions of the hippocampus
Source: PLoS One. 2026 Mar 19;21(3):e0344593. doi: 10.1371/journal.pone.0344593 (PMC13001954; doi:10.1371/journal.pone.0344593)

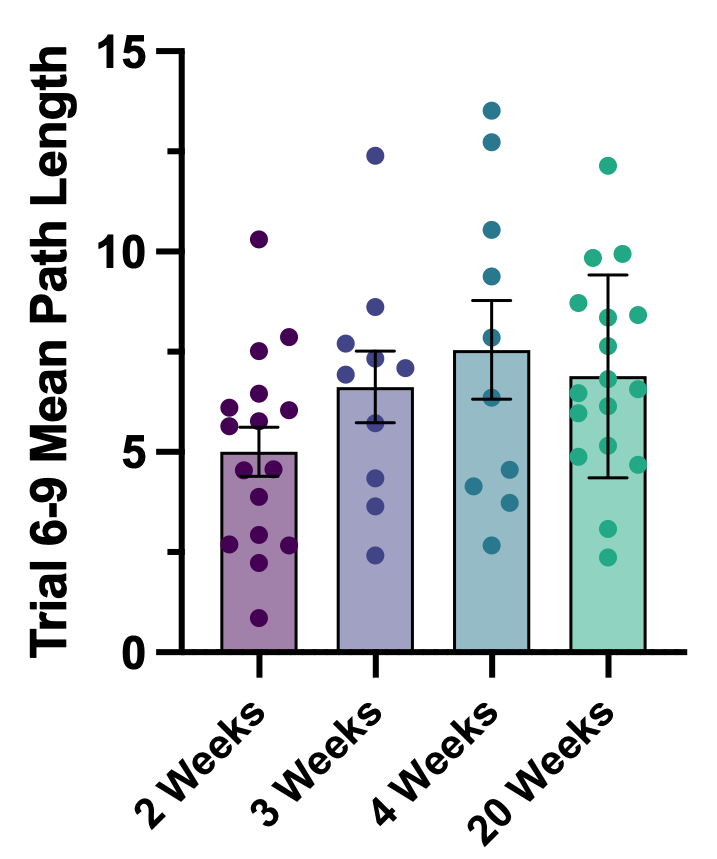

Supplement: S1 Fig — Mean (±SEM) path length for each subject’s trial 6–9 average. A one-way ANOVA revealed no significant differences between the different surgery–behavioural testing intervals (F3,49 = 2, P = .126). (TIFF) [file pone.0344593.s001.tiff]

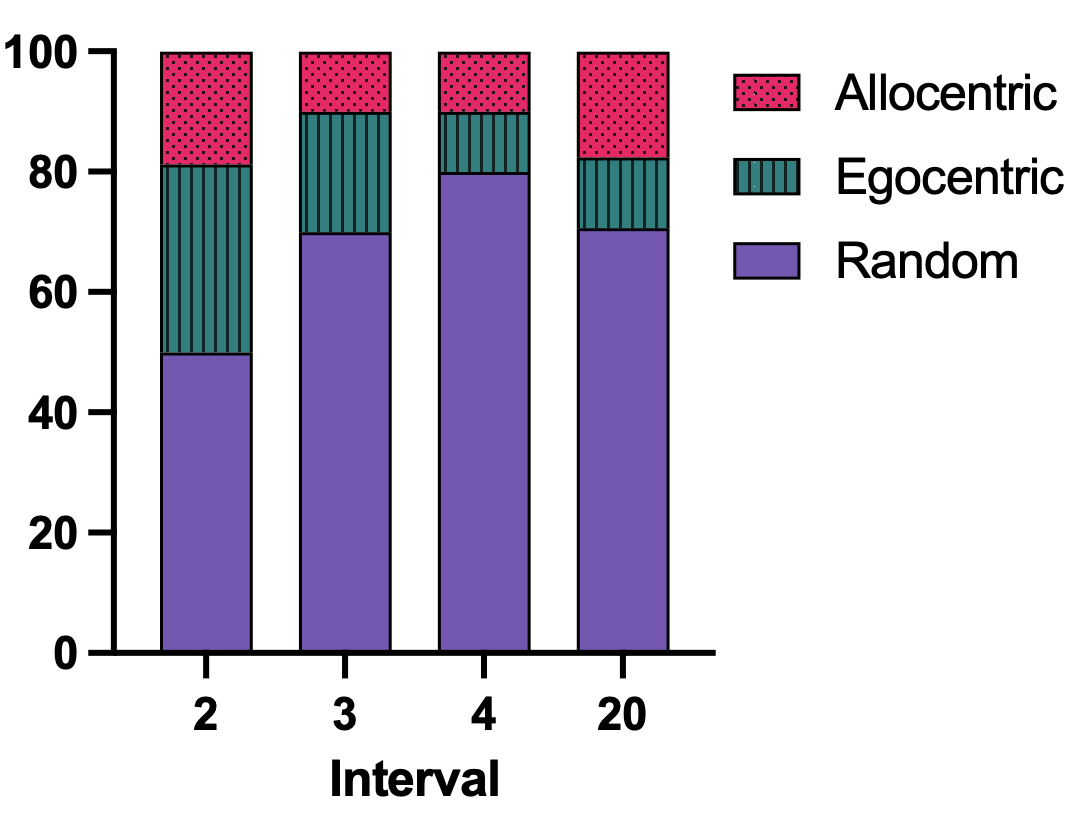

Supplement: S2 Fig — Percentage of rats assigned to allocentric, egocentric, and random subgroups for each interval. Chi-square tests for independence determined that the distribution of subgroups did not significantly differ between intervals (A-HPC χ²3 = 2, P = .572; E-HPC χ²3 = 3.6, P = .308; R-HPC χ²3 = 1.69, P = .640). (TIFF) [file pone.0344593.s002.tiff]
