# Supplementary material for: Evidence of allocentric spatial learning in male rats with large lesions of the hippocampus
Source: PLoS One. 2026 Mar 19;21(3):e0344593. doi: 10.1371/journal.pone.0344593 (PMC13001954; doi:10.1371/journal.pone.0344593)
Supplement: S1 Table — (DOCX) [file pone.0344593.s003.docx]

**S1 Table. Stereotaxic injection coordinates relative to bregma (mm) and volume of NMDA+TTX infused at each site.**

| Anteroposterior (AP) | Mediolateral (ML) | Dorsoventral (DV) | Infusion Volume (μL) |
| --- | --- | --- | --- |
| -3.0 | ±1.5 | -3.6 | 0.3 |
| -4.0 | ±3.0 | -4.0 | 0.3 |
| -4.9 | ±3.0 | -4.0 | 0.3 |
| -4.9 | ±5.2 | -7.2 | 0.3 |
| -5.7 | ±4.4 | -4.4 | 0.3 |
| -5.7 | ±5.4 | -7.3 | 0.4 |
| -5.7 | ±5.4 | -6.0 | 0.4 |
