# Supplementary material for: Evidence of allocentric spatial learning in male rats with large lesions of the hippocampus
Source: PLoS One. 2026 Mar 19;21(3):e0344593. doi: 10.1371/journal.pone.0344593 (PMC13001954; doi:10.1371/journal.pone.0344593)
Supplement: S2 Table — (DOCX) [file pone.0344593.s004.docx]

**S2 Table. Rater agreement on swim path classification.**

| Comparison | Category | Agreement (%) |
| --- | --- | --- |
| Rater 1 vs Rater 2 | Total | 87 |
|  | Allocentric | 86 |
|  | Egocentric | 81 |
|  | Random | 90 |
| Rater 1 vs RODA | Total | 77 |
|  | Allocentric | 99 |
|  | Egocentric | 59 |
|  | Random | 76 |
| Rater 2 vs RODA | Total | 74 |
|  | Allocentric | 94 |
|  | Egocentric | 51 |
|  | Random | 74 |
